# Tension-type headache patients’ brain responses to instant acupuncture stimulation: a functional magnetic resonance imaging study

**DOI:** 10.3389/fneur.2025.1633823

**Published:** 2025-09-02

**Authors:** Xinyue Zhang, Wenhua Wang, Siyuan Tao, Jun Zhou, Nannan Jiang, Xinling Li, Shengjie Hu, Weiming Luo, Nuo Chen, Yu Fang, Huabin Zheng, Fanrong Liang, Fang Zeng, Zhengjie Li

**Affiliations:** ^1^Acupuncture and Tuina School, Chengdu University of Traditional Chinese Medicine, Chengdu, China; ^2^Key Laboratory of Acupuncture for Senile Disease (Chengdu University of TCM), Ministry of Education, Chengdu, China; ^3^Acupuncture and Brain Research Center, Chengdu University of Traditional Chinese Medicine, Chengdu, China; ^4^Department of Radiology, The Second Affiliated Hospital of Chengdu Medical College, Nuclear Corportation 416 Hosptital, Chengdu, China; ^5^Department of Neurology, Hospital of Chengdu University of Traditional Chinese Medicine, Chengdu, China; ^6^Department of Acupuncture and Moxibustion, Hospital of Chengdu University of Traditional Chinese Medicine, Chengdu, China

**Keywords:** tension-type headache, acupuncture, instant effects, functional magnet resonance imaging, multiple analysis methods

## Abstract

**Objectives:**

To investigate the effects of instant acupuncture stimulation on brain activity in patients with tension-type headache (TTH) compared to healthy controls (HCs).

**Methods:**

Thirty-six TTH patients and thirty-six HCs participated in this study. Both groups underwent resting-state and acupuncture-state functional magnetic resonance imaging (fMRI) scans. The amplitude of low-frequency fluctuation (ALFF), fractional ALFF (fALFF), and regional homogeneity (ReHo) were used to assess spontaneous brain activity of participants. Additionally, participants’ acupuncture sensation scores during stimulation were recorded, and brain activity differences between TTH patients and HCs with similar sensations (with predominately *Deqi* sensation or acute pain) were compared. This study was officially registered on the Chinese Clinical Trial Registry (ChiCTR) as a sub-study of the parent clinical trial (No. ChiCTR2100042915).

**Results:**

Both groups showed activation in the superior frontal gyrus, supramarginal gyrus, and precuneus, along with deactivation in the precentral/postcentral gyrus, inferior occipital gyrus, lingual gyrus, and superior temporal gyrus during acupuncture stimulation. Notably, TTH patients also exhibited increased activity in the anterior cingulate cortex and caudate nucleus, as well as decreased activity in the parietal operculum. TTH patients and HCs with acute pain sensations demonstrated similar spontaneous functional brain activity in the precentral/postcentral gyrus, lingual gyrus, and cuneus/precuneus. Furthermore, HCs with pronounced *Deqi* sensations exhibited functional changes in the precentral/postcentral gyrus, whereas no such changes were observed in the TTH patients.

**Conclusion:**

The modification of functional activity in the sensorimotor system, default mode network (DMN), and visual network (VN) during acupuncture stimulation suggest common brain responses in both TTH patients and HCs. In addition, acupuncture at *Siguan* acupoints could extensively regulate the limbic system in TTH patients, and showed targeted modulation in the abnormal brain regions of the ACC and caudate nucleus, which are closely related to the regulation of pain emotions and cognition.

**Clinical trial registration:**

This study was officially registered on the Chinese Clinical Trial Registry (ChiCTR, https://www.chictr.org.cn/) as a sub-study of the parent clinical trial (No. ChiCTR2100042915).

## Introduction

Tension-type headache (TTH) is the most prevalent neurological disorder worldwide (1), characterized by a bilateral, pressing, or tightening headache, which is generally mild to moderate in intensity and not aggravated by routine physical activity (2). TTH has become a major global public health problem, imposing an unnoticeable burden on patients, their families, and society. The pathophysiology of TTH is multifactorial, involving both peripheral and central mechanisms. In recent years, studies have revealed that TTH patients exhibit functional (3–5) and structural (6–8) brain abnormalities detected by magnetic resonance imaging (MRI), with the affected regions being associated with pain integration and processing. These findings provided valuable insights for further exploration of TTH mechanisms.

Acupuncture, an important component of complementary and alternative medicine, has been proven safe and effective in treating TTH (9, 10), and it is widely used in clinical practice in China. Numerous studies have indicated that acupuncture can activate specific regions of central nervous system (CNS) and complete the integration and transformation of acupuncture signals in the CNS (11–13). However, little is known about the characteristics of TTH patients’ brain responses to acupuncture stimulation. Investigating how acupuncture signals are processed in the CNS in TTH patients may further deepen our cognization of the disease mechanism and the specific effects of acupuncture on TTH.

Functional magnetic resonance imaging (fMRI) is a non-invasive neuroimaging technique widely used in chronic pain, psychiatry, psychology, neurology and acupuncture researches (14–16). The amplitude of low-frequency fluctuations (ALFF) measures the amplitude of a specific frequency band (typically 0.01–0.1 Hz) of BOLD oscillations. Fractional ALFF (fALFF) normalizes ALFF with the amplitude of the entire frequency spectrum to show the relative contribution of low-frequency fluctuations within the entire pattern, minimizing the impact of ventricular and blood flow noise on ALFF detection (17). Regional homogeneity (ReHo) estimates the local synchronization of band-filtered BOLD signals between a specific voxel and its neighbors (18). ALFF, fALFF, and ReHo all reflect local characteristics in spatially discrete brain regions and offer a progressive and complementary relationship in representing different aspects of brain function. Therefore, synthesizing these indicators is valuable for exploring the multi-spatial voxel-scale modulation mechanisms of instant acupuncture needling stimulation on the CNS in TTH patients.

Based on the above, this study combines three functional neuroimaging analysis methods—ALFF, fALFF, and ReHo—to investigate the CNS responses to acupuncture at the *Siguan* acupoints in both TTH patients and healthy controls (HCs). Given the CNS abnormalities associated with TTH, we hypothesize that TTH patients will exhibit both similar and distinct patterns of brain activity in response to acupuncture stimulation, compared to HCs. In addition, we also tried to explore the specific effects of composite *Deqi* sensations or acute pain on brain activity to deepen our understanding of how acupuncture influences central pain processing in TTH.

## Materials and methods

### Participants

Thirty-six TTH patients and thirty-six HCs were participated in this study. TTH patients were asked to keep recording their headache diaries for at least 1 month before the study, and they must be headache-free for at least 72 h before and after the fMRI scans.

TTH patients were included if they: (1) were 18–65 years old and right-handed; (2) met the diagnosis of TTH based on *the International Classification of Headache Disorders, 3rd edition* in 2018 (2); (3) had a history of TTH for more than 6 months; (4) were able to complete the headache diary independently; (5) had no fear of acupuncture, had no previous history of acupuncture fainting, and agreed to accept intradermal needling; and (6) signed the informed consents by themselves.

TTH patients were excluded if they: (1) suffered from other types of primary or secondary headache; (2) had any other neurological or psychiatric disorder; (3) were pregnant or breast-feeding; (4) had the contraindications for MRI or acupuncture; or (5) were deemed inappropriate to participate in the trial by the investigators, including poor compliance.

All the patients were recruited from the outpatient department of the Teaching Hospital of Chengdu University of Traditional Chinese Medicine (TCM), Elite Clinic of Chengdu University of TCM, and campus of Chengdu University of TCM during the period from January 2021 to October 2023. The HC group was recruited from the campus and community to match the age, gender, years of education and handedness of the TTH group, and these volunteers had never been diagnosed with head trauma, alcohol/drug abuse, and neurological or psychiatric disorders. All participants signed a written informed consent form before the study, as required by the ethics committee (Hospital of Chengdu University of Traditional Chinese Medicine, Ethics Approval Number: 2020KL-058). This study was officially registered on the Chinese Clinical Trial Registry (ChiCTR) as a sub-study of the parent clinical trial (No. ChiCTR2100042915).

### Acupuncture stimulation procedure

**Figure 1:**
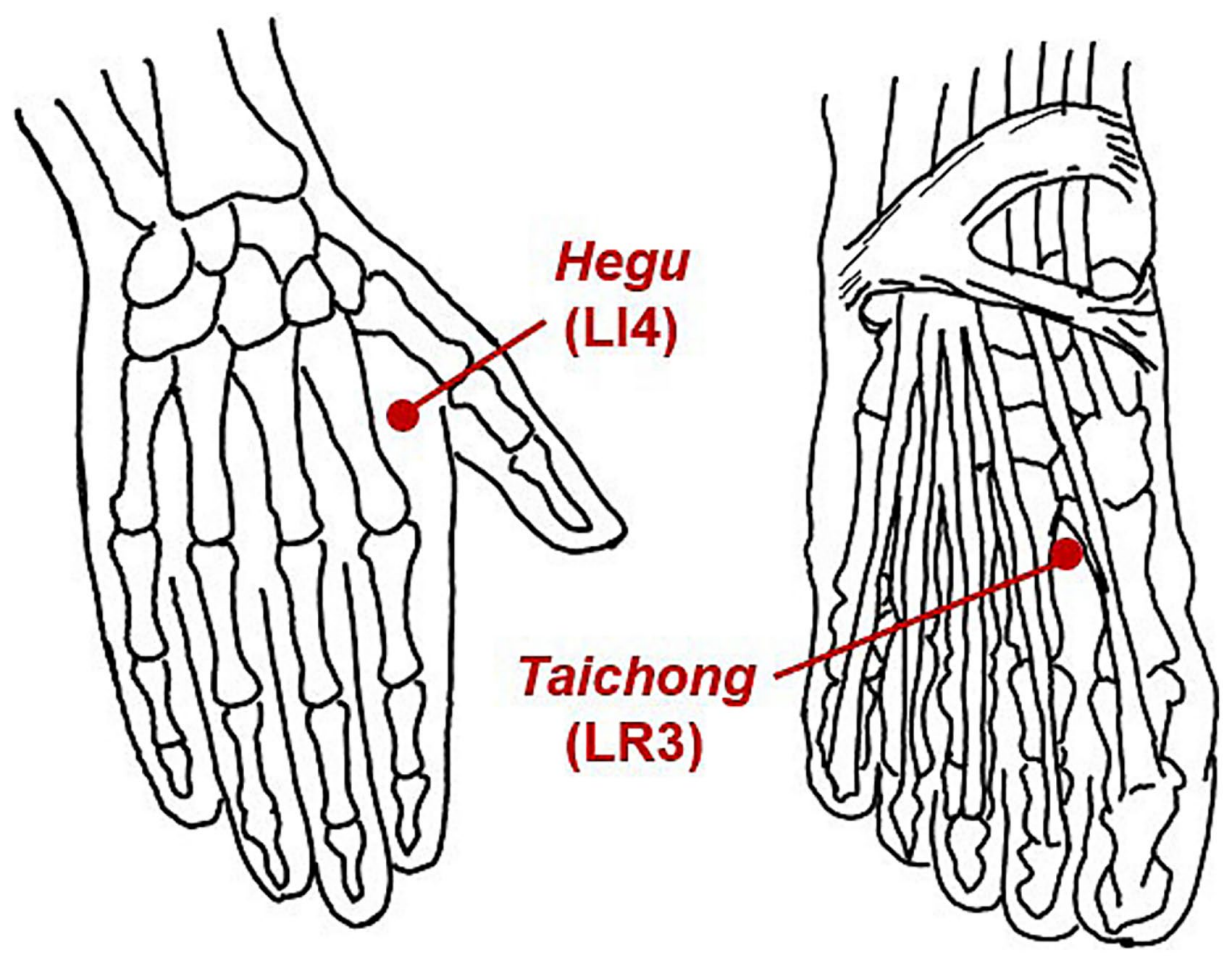
The locations of *Siguan* acupoints. *Siguan* acupoints are composed of bilateral *Hegu (LI4)* and bilateral *Taichong (LR3)*. *Hegu* (LI4) is on the dorsum of the hand, the midway between the 1st and 2nd metacarpal bones, approximately in the middle of the 2nd metacarpal bone on the radial side. *Taichong* (LR3) is on the dorsum of the foot, in the depression distal to the junction of the 1st and 2nd metatarsal bones.

**Figure 2:**
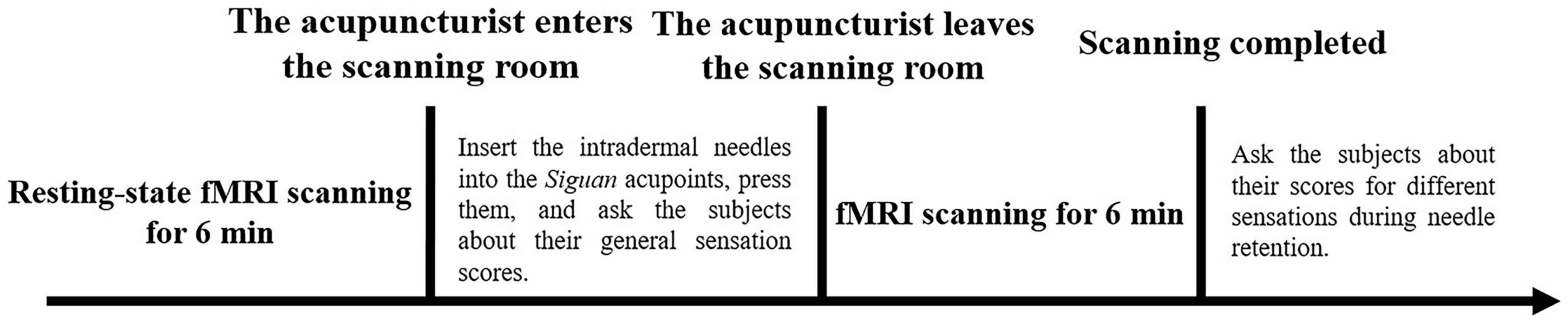
The flowchart displaying the fMRI scanning.

All participants underwent intradermal acupuncture stimulation after a 6-min resting-state fMRI scan. When the 6-min resting-state fMRI scan was finished, the acupuncturist entered the MRI scanning room. After disinfecting the skin at the local acupoints, the acupuncturist inserted four intradermal acupuncture needles (HUANQIU disposable press needles; registration number 20162270591; size 0.25 × 1.30 mm) into participants’ *Siguan* acupoints [bilateral *Hegu* (LI4) and bilateral *Taichong* (LR3)] and then pressured them. Each acupoint was pressed 10 times in the order of the left *Hegu* (LI4), the left *Taichong* (LR3), the right *Taichong* (LR3), and the right *Hegu* (LI4), with a pressing depth 8–10 mm for LI4 and 5–8 mm for LR3. At the end of pressing, the subjects were asked to score their general acupuncture needling sensation. The sensation score ranged from 0 to 10, with 0 indicating no sensation and 10 representing the maximum sensation. The four intradermal needles were then preserved in the four acupoints while the 6-min acupuncture state fMRI scan was proceeding. After the fMRI scan, participants were acquired to recall the needling sensations experienced during the acupuncture stimulation fMRI in time, which included sensations associated with the category of *Deqi* (soreness, numbness, distension, heaviness, or other special sensations) and the sensation with the category of acute pain. Each sensation score was also evaluated with scores from 0 to 10. Prior to fMRI scanning, all participants received standardized training from a researcher to help differentiate *Deqi* sensations from acute pain, which included verbal descriptions and visual analog scale practice to ensure consistent reporting. The locations of *Siguan* acupoints are shown in Figure 1, and the details of the needling and fMRI scanning procedures were displayed in Figure 2.

### Clinical data acquisition

Before the fMRI scan, all TTH patients were required to keep headache diaries for a month to record their headache episodes. The duration of TTH, frequency of headache attacks, number of days with headache, headache lasting days, maximum and average headache visual analogue scale (VAS) scores were recorded. The VAS score is a 10-cm scale, with 0 indicating no pain and 10 representing the worst pain, which is a commonly used tool for evaluating headache intensity.

### MRI data acquisition

MRI data were acquired from two centers with the same settings of parameters. One center used a 3.0 T magnetic resonance scanner (GE Discovery 750; Milwaukee, WI, United States) with a 16-channel phase-array head coil at the University of Electronic Science and Technology of China, while the other used a 3.0 T magnetic resonance scanner (GE SIGNA Architect; USA) with a 19-channel phase-array head coil at the Second Affiliated Hospital of Chengdu Medical College. Participants were instructed to lie supine on the examination bed, keep their heads still, stay conscious while avoiding wandering thoughts, and keep their eyes closed and ears plugged throughout the entire scan.

Firstly, the three-dimensional T1-weighted (3D-T1) imaging was performed to obtain high-resolution structural images for each participant with a voxel size of 1 mm^3^, using a spoiled gradient-recalled sequence (repetition time = 6.008 ms; echo time = 1.7 ms; field of view = 256 × 256 mm^2^; data matrix = 256 × 256). Then, the resting-state and the acupuncture-state BOLD-fMRI signals were obtained using the same gradient-recalled echo planar imaging (31 contiguous slices, slice thickness = 5 mm; repetition time = 2,000 ms; echo time = 30 ms; flip angle = 90^°^; field of view = 240 × 240 mm^2^; data matrix = 64 × 64; total volumes = 205).

### Statistical analysis of clinical data

Clinical data were analyzed using SPSS 26.0 statistical software (IBM, Armonk, NY, United States). Continuous variables that followed a normal distribution were described as means with standard deviations, while continuous variables that did not follow a normal distribution were represented by medians and interquartile ranges. Categorical variables were expressed as number and proportions. The two-sample t-test and chi-square test were used to compare the baseline data between TTH patients and HCs, and statistical significance was set at *p* < 0.05.

### Statistical analysis of MRI data

The fMRI data were preprocessed using MATLAB R2021a (Mathworks, Natick, MA, United States) and DPABI V8.0.^1^ During the preprocessing process, the data were first converted from DICOM format to NIFTI format, and 10 time points were removed to ensure the stability of the signal. Then, the slice time and head motion were corrected. Subjects with head motion or translation greater than 2.0° or 2.0 mm were excluded. The spatial normalization parameter was set to 3mm × 3mm × 3mm. ALFF and fALFF were first smoothed with a 6 mm full width at half maximum (FWHM) and then analyzed, while ReHo was initially analyzed and subsequently smoothed with an 8 mm FWHM. Then, the images were detrended and bandpass-filtered between 0.01 Hz and 0.08 Hz. The square root of the power spectrum in the range of 0.01 to 0.08 Hz was calculated and averaged, with the average square root value representing the ALFF value. The fALFF value was calculated by dividing the power within the ALFF by the total power in the entire measurable frequency range. Subsequently, ReHo was calculated by determining the Kendall concordance coefficient of a given voxel and its 26 neighboring voxels in the same time series to generate a ReHo map for each subject. As the fMRI data were acquired from two different sites, we harmonized the data using the shift correction algorithm (SMA) to reduce the influence of sites effects on the results (19). SMA exhibits robust capability in mitigating site-specific variability while preserving biologically relevant signals, demonstrating high identifiability, excellent test–retest reliability, and remarkable stability. The data were analyzed using SPM12 (Wellcome Department of Imaging Neuroscience, London, UK; http://www.fil.ion.ucl.ac.uk/spm) software. We applied paired t-tests to compare resting-state and acupuncture state values of ALFF, fALFF, and ReHo images in each group. Family-wise error correction (FWE) with a threshold of *p* < 0.05, and cluster sizes >30 were adopted for all analyses (20, 21).

## Results

### Studies demographics

The baseline demographic characteristics of all participants and the clinical data of patients were summarized in Table 1. There were no significant differences between the two groups in gender, age, height, weight (*p* > 0.05).

**Table 1:** Baseline demographic and clinical characteristics of TTH patients and HCs.

Item	TTH	HC		*p* value
Gender (male/female)	8/28	11/25	χ^2^ = 0.643	0.422
Handedness (right)	36	36	-	-
Age (y)	25.39 ± 6.26	24.83 ± 2.85	*t*’ = 0.484	0.630
Height (cm)	161.44 ± 10.99	165.22 ± 7.72	*t* = −1.688	0.096
Weight (kg)	55.95 ± 9.00	59.94 ± 11.01	*t* = −1.685	0.096
Duration of TTH (month)	66.63 ± 53.89	-	-	-
Frequency of headache attacks per month	5(3.25, 7.75)	-	-	-
Number of days with headache per month	5(4, 7.75)	-	-	-
Headache last days per month	1.46 (0.83, 2.45)	-	-	-
Maximum VAS scores	5.46 ± 1.74			
Average VAS scores	4.27 ± 1.21	-	-	-

TTH, tension-type headache; HC, healthy control; VAS, visual analogue scale.

### TTH patients and HCs’ brain activities after instant intradermal acupuncture stimulation

There was no difference (*p* > 0.05) in general sensation between TTH patients (4.11 ± 1.01) and HCs (3.86 ± 0.87) during instant intradermal acupuncture stimulation (*t* = 1.128, *p* = 0.263).

**Figure 3:**
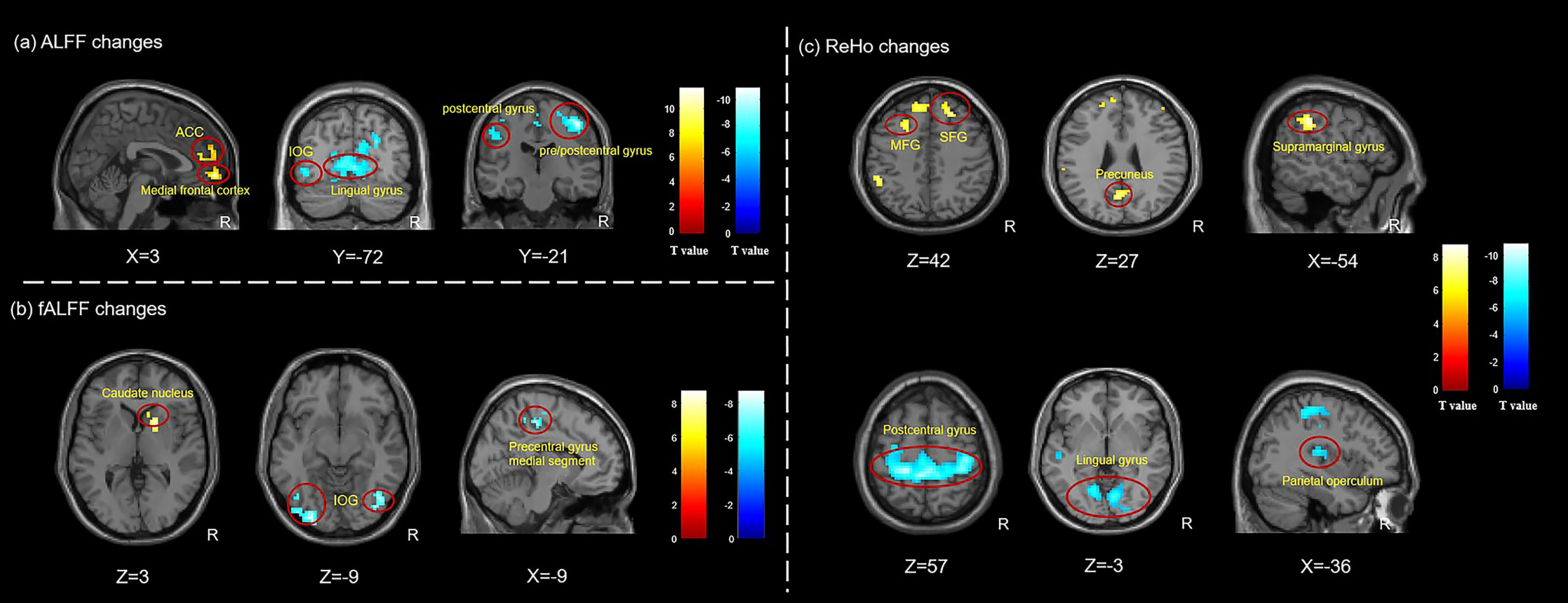
Changes of brain activities in TTH patients after instant intradermal acupuncture stimulation. **(a)** ALFF changes in TTH patients during instant acupuncture needling stimulation; **(b)** fALFF changes in TTH patients during instant acupuncture needling stimulation; **(c)** ReHo changes in TTH patients during instant acupuncture needling stimulation. ALFF, amplitude of low-frequency fluctuation; fALFF, fractional amplitude of low-frequency fluctuation; ReHo, regional homogeneity; TTH, tension-type headache; ACC, anterior cingulate cortex; IOG, inferior occipital gyrus; MFG, middle frontal gyrus; SFG, superior frontal gyrus. The analyses were FWE corrected, with *p* < 0.05.

During instant intradermal acupuncture stimulation, several changes in brain functional activities were detected in TTH patients. The ALFF values decreased in the bilateral lingual gyrus, the bilateral postcentral gyrus, the right precentral gyrus, and the left inferior occipital gyrus, while they increased in the right anterior cingulate cortex (ACC) and the right medial frontal cortex. The fALFF values decreased in the bilateral precentral gyrus medial segment and the bilateral inferior occipital gyrus, while they increased in the right caudate nucleus. The ReHo values decreased in the bilateral postcentral gyrus, the bilateral lingual gyrus, the left parietal operculum, and the left superior temporal gyrus, while they increased in the bilateral middle frontal gyrus, the right superior frontal gyrus, the left supramarginal gyrus, and the left precuneus (Figure 3; Table 2).

**Table 2:** ALFF, fALFF, and ReHo changes in TTH patients during instant acupuncture stimulation.

State	Brain regions	Hemi	Cluster size	MNI coordinates			T value
				X	Y	Z	
ALFF							
Acupuncture state < Resting state	Lingual gyrus	L/R	1,125	−9	−78	−9	−8.21
	Precentral/Postcentral gyrus	R	246	48	−21	51	−10.84
	Postcentral gyrus	L	108	−18	−30	60	−8.86
	Postcentral gyrus	L	74	−51	−18	36	−8.44
	Inferior occipital gyrus	L	30	−45	−69	−3	−8.18
Acupuncture state > Resting state	Anterior cingulate cortex	R	72	3	45	12	8.30
	Medial frontal cortex	R	42	3	60	−6	11.62
fALFF							
Acupuncture state < Resting state	Precentral gyrus medial segment	L/R	152	−9	−27	48	−7.58
	Inferior occipital gyrus	R	116	45	−75	−6	−7.89
	Inferior occipital gyrus	L	153	−39	−84	−12	−7.74
Acupuncture state > Resting state	Caudate nucleus	R	44	18	6	0	7.97
ReHo							
Acupuncture state < Resting state	Lingual gyrus	L/R	656	9	−72	−3	−8.13
	Postcentral gyrus	R	42	57	−6	24	−6.40
	Postcentral gyrus	L	1,189	−6	−39	57	−9.56
	Lingual gyrus	L	195	−12	−75	−9	−7.38
	Parietal operculum	L	38	−36	−24	15	−6.83
	Superior temporal gyrus	L	37	−54	−15	−9	−6.11
Acupuncture state > Resting state	Middle frontal gyrus	R	46	45	42	12	6.94
	Superior frontal gyrus	R	41	21	36	42	7.17
	Middle frontal gyrus	L	375	−24	21	42	6.82
	Supramarginal gyrus	L	70	−54	−39	36	7.96
	Precuneus	L	52	0	−63	27	7.23

FWE corrected, p < 0.05, cluster size>30. ALFF, amplitude of low-frequency fluctuation; fALFF, fractional amplitude of low-frequency fluctuation; ReHo, regional homogeneity; TTH, tension-type headache; MNI, Montreal neurological institute; L, left; R, right.

**Figure 4:**
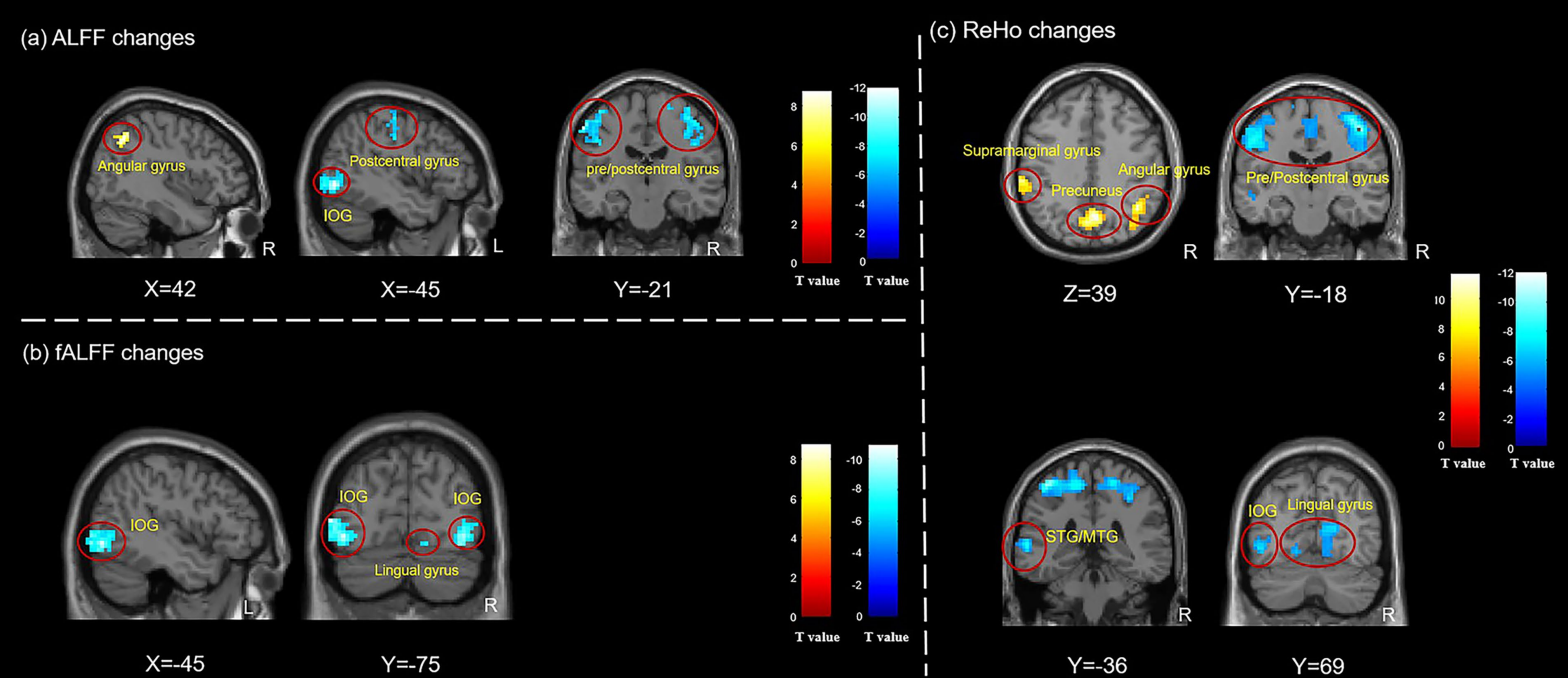
Changes of brain activities in HCs patients after instant intradermal acupuncture stimulation. **(a)** ALFF changes in HCs during instant acupuncture needling stimulation; **(b)** fALFF changes in HCs during instant acupuncture needling stimulation; **(c)** ReHo changes in HCs during instant acupuncture needling stimulation. ALFF, amplitude of low-frequency fluctuation; fALFF, fractional amplitude of low-frequency fluctuation; ReHo, regional homogeneity; HCs, healthy controls; IOG, inferior occipital gyrus; STG, superior temporal gyrus; MTG, middle temporal gyrus. The analyses were FWE corrected, with *p* < 0.05.

During instant intradermal acupuncture stimulation, variations in brain functional activities were also found in HCs. The ALFF values decreased in the bilateral precentral/postcentral gyrus, the left inferior occipital gyrus, the left lingual gyrus, and the left precentral gyrus medial segment, while they increased in the right angular gyrus. The fALFF values decreased in the bilateral inferior occipital gyrus and the right lingual gyrus. The ReHo values decreased in the bilateral precentral/postcentral gyrus, the bilateral lingual gyrus, the left superior/middle temporal gyrus, and the left inferior occipital gyrus, while they increased in the bilateral precuneus, the right angular gyrus, the left supramarginal gyrus, and the left superior frontal gyrus (Figure 4; Table 3).

**Table 3:** ALFF, fALFF, and ReHo changes in HCs during instant acupuncture stimulation.

State	Brain regions	Hemi	Cluster size	MNI coordinates			T value
				X	Y	Z	
ALFF							
Acupuncture state < Resting state	Precentral/Postcentral gyrus	R	139	39	−18	57	−8.41
	Precentral gyrus	R	40	12	−30	63	−6.30
	Inferior occipital gyrus	L	763	−45	−72	−6	−12.04
	Postcentral/Precentral gyrus	L	113	−57	−18	48	−11.51
	Lingual gyrus	L	68	−9	−78	−9	−8.50
	Precentral gyrus medial segment	L	45	−3	−27	51	−8.04
Acupuncture state > Resting state	Angular gyrus	R	47	42	−57	45	9.73
fALFF							
Acupuncture state < Resting state	Lingual/Inferior occipital gyrus	R	216	21	−60	−12	−10.69
	Inferior occipital gyrus	L	264	−45	−75	−6	−9.80
Acupuncture state > Resting state	No results						
ReHo							
Acupuncture state < Resting state	Postcentral/Precentral gyrus	L/R	1,478	−39	−33	63	−13.17
	Lingual gyrus	R	418	18	−57	−6	−9.56
	Superior/Middle temporal gyrus	L	146	−60	−36	6	−9.19
	Inferior occipital gyrus	L	58	−45	−72	−3	−8.14
	Lingual gyrus	L	38	−24	−60	−9	−6.56
Acupuncture state > Resting state	Precuneus	L/R	287	3	−63	39	10.73
	Angular gyrus	R	115	39	−69	39	7.01
	Supramarginal gyrus	L	103	−63	−30	36	10.20
	Superior frontal gyrus	L	69	−18	18	54	6.26

FWE corrected, p < 0.05, cluster size>30. ALFF, amplitude of low-frequency fluctuation; fALFF, fractional amplitude of low-frequency fluctuation; ReHo, regional homogeneity; MNI, Montreal neurological institute; HCs, healthy controls; L, left; R, right.

### Brain functional activities influenced by different needling sensation

**Figure 5:**
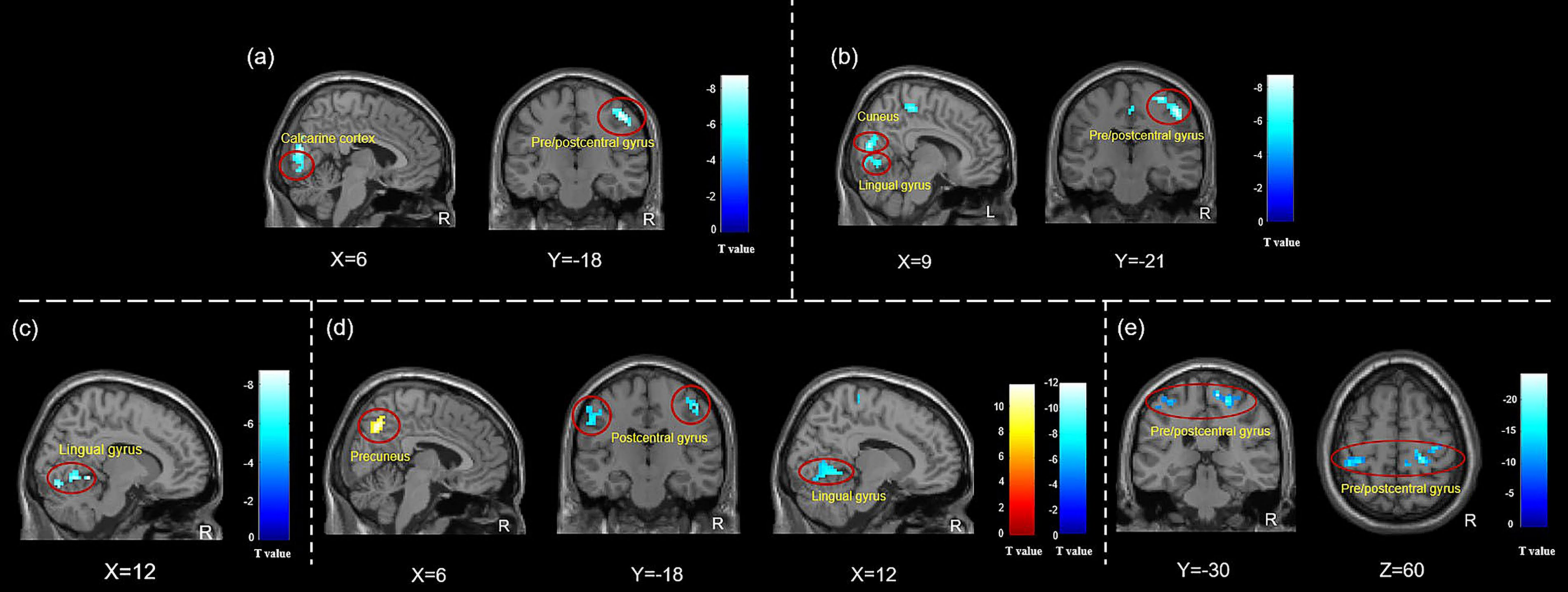
ALFF and ReHo changes in subjects with different needling sensations during instant acupuncture needling stimulation. **(a)** ALFF changes in TTH patients with acute pain sensation during instant acupuncture; **(b)** ReHo changes in TTH patients with acute pain sensation during instant acupuncture; **(c)** ALFF changes in HCs with acute pain sensation during instant acupuncture; **(d)** ReHo changes in HCs with acute pain sensation during instant acupuncture; **(e)** ReHo changes in HCs with composite *Deqi* sensation during instant acupuncture. Abbreviation: ALFF, amplitude of low-frequency fluctuation; ReHo, regional homogeneity. The analyses were FWE corrected, with *p* < 0.05.

Twenty-three TTH patients reported higher levels of acute pain scores compared to composite *Deqi* sensation, while ten TTH patients had higher scores for composite *Deqi* sensation, and three patients had same scores for composite *Deqi* sensation and acute pain. In the twenty-three patients with more pronounced acute pain sensation, the results showed that the ALFF values were decreased in the bilateral calcarine cortex and the right precentral/postcentral gyrus, while the ReHo values were decreased in the right precentral/postcentral gyrus, the right cuneus and the right lingual gyrus. In contrast, no changes in brain functional activities were observed in the 10 patients with stronger composite *Deqi* sensation. No results were found using fALFF analysis. (Figure 5, Table 4).

**Table 4:** ALFF and ReHo changes in subjects with different needling sensations during instant acupuncture needling stimulation.

Indicator	Brain regions	Hemi	Cluster size	MNI coordinates			T value
				X	Y	Z	
TTH patients with acute pain sensation							
ALFF	Calcarine cortex	L/R	189	6	−78	3	−7.36
	Precentral/Postcentral gyrus	R	65	48	−24	51	−9.37
ReHo	Precentral/Postcentral gyrus	R	376	48	−21	48	−9.50
	Cuneus	R	100	9	−81	15	−8.73
	Lingual gyrus	R	43	9	−72	−3	−6.97
TTH patients with composite *Deqi* sensation							
ALFF	No results						
ReHo	No results						
HCs with acute pain sensation							
ALFF	Lingual gyrus	R	55	12	−48	−6	−9.31
ReHo	Precentral/Postcentral gyrus	R	126	21	−30	60	−8.49
	Lingual gyrus	R	116	12	−54	−6	−8.82
	Postcentral gyrus	L	211	−39	−33	63	−11.24
	Precuneus	R	91	6	−63	42	10.47
HCs with composite *Deqi* sensation							
ALFF	No results						
ReHo	Precentral/Postcentral gyrus	R	96	12	−30	66	−24.24
	Postcentral gyrus	L	40	−42	−36	60	−14.96

FWE corrected, *p* < 0.05, cluster size>30. ALFF, amplitude of low-frequency fluctuation; ReHo, regional homogeneity; MNI, Montreal neurological institute; HCs: healthy controls; L, left; R, right.

Twenty-one HCs experienced higher levels of acute pain scores in comparison to the composite *Deqi* sensation, whereas eleven HCs exhibited higher scores for the composite *Deqi* sensation, and four HCs had the same scores for composite *Deqi* sensation and acute pain. Decreased ALFF and ReHo values in the right lingual gyrus, decreased ReHo values in the bilateral postcentral gyrus and the right precentral gyrus, and the increased values in the precuneus were observed in HCs with pronounced acute pain sensation. Decreased ReHo values in the bilateral postcentral gyrus and the right precentral gyrus were detected in HCs with stronger composite *Deqi* sensation. No results were found using fALFF analysis. (Figure 5, Table 4).

## Discussion

In this study, we integrated three functional neuroimaging analysis methods to investigate the modulated effects of instant acupuncture stimulation on brain activities in patients with TTH and HCs. Additionally, we investigated the changes in brain activity of the two groups with *Deqi* sensations and acute pain, respectively.

During acupuncture stimulation, both TTH group and HC group showed activated modulations in the superior frontal gyrus, the supramarginal gyrus, and the precuneus, along with deactivated variations in the precentral/postcentral gyrus, the inferior occipital gyrus, the lingual gyrus, and the superior temporal gyrus. Most of these regions are related to the sensorimotor system, the default mode network (DMN), and the visual network (VN). In addition to aforementioned brain regions, TTH patients specially exhibited increased activity in the ACC and caudate nucleus, as well as decreased activity in the parietal operculum. During acupuncture stimulation, both TTH patients and HCs with acute pain exhibited spontaneous functional brain activity in regions associated with the sensorimotor system and DMN, such as the precentral/postcentral gyrus and the lingual gyrus and cuneus/precuneus. In individuals with pronounced *Deqi* sensation, HCs showed alterations in functional brain activity in the precentral/postcentral gyrus, while no changes were found in TTH patients.

### The modification of functional activities in the sensorimotor system, DMN and VN is a common central response characteristic observed in both TTH patients and HCs during acupuncture

Both TTH patients and HCs exhibited altered functional activity in the bilateral precentral gyrus and postcentral gyrus during acupuncture at *Siguan* acupoints. According to cortical topography, the changed brain regions were mainly located in areas governing the movement and sensory functions of the foot, hand and forehead. It may due to location of the acupoints stimulated and their remote therapeutic effects (22, 23). Additionally, ALFF, fALFF, and ReHo values were changed in several brain regions of the DMN (including the superior frontal gyrus, the precuneus, and the superior temporal gyrus) in both TTH patients and HCs during acupuncture. DMN is more active when individuals are not concentrating on the external environment, and the interruption of the activity of attention-demanding tasks will lead to suppression of the system (24). In addition to being involved in switching networks, various nodes of the DMN are associated with cognitive functions (25, 26). Research has suggested that prominent stimulus-induced external events drive network switches, shaping the dynamic role of the DMN in cognition (27). The alterations in functional activity within the DMN-associated brain regions during acupuncture at *Siguan* acupoints might be closely related to the switching of subjects’ attention in response to external stimulation.

Furthermore, the VN (the inferior occipital gyrus and the lingual gyrus) was altered during intradermal acupuncture needling in both TTH patients and HCs. The occipital lobe is the central hub of the visual cortex, primarily responsible for processing visual information (28). Some studies have suggested that stimulating acupoints related to vision can specifically activate the visual cortex, which may be due to the specificity of acupoints (29). However, other studies held the converse opinion (30, 31). A PET study found that activation of the virtual cortex was triggered during the perception of heat pain (47°C vs. 34°C), while the perception of warmth (40°C vs. 34°C) did not lead to this phenomenon (32). In an fMRI study with participants’ eyes and ears closed, the visual cortex was activated in subjects experiencing rectal pain (33). In this study, it was detected that the instant acupuncture of the *Siguan* acupoints led to negative activation in the visual cortex in participants, suggesting that this may not relate to the specific effects of the acupoints, but rather to the sensory cross-modality interaction caused by somatic-visceral sensory stimulation (34).

### Acupuncture needling at *Siguan* acupoints can extensively regulate the limbic system in TTH patients

Compared to the alterations in spontaneous functional brain activity induced by acupuncture stimulation at the *Siguan* acupoints in HCs, TTH patients not only showed distinct changes in the right ACC, the right caudate nucleus, and the left parietal operculum, but also exhibited more extensive functional changes in the frontal lobe. Obviously, acupuncture at the *Siguan* acupoints may have a broader effect on brain areas within the limbic system in TTH patients.

Recent neuroimaging studies have established the critical involvement of the limbic system in pain chronification, pain cognition, affection and nociception modulation (35, 36). Of particular relevance to TTH, the ACC - a key node in the “pain matrix,” demonstrates gray matter volume changes in TTH patients (6, 7). Our findings reveal that acupuncture can specifically modulate ACC activity, potentially normalizing its impaired function in emotional pain processing (37). Notably, we observed acupuncture-induced enhancement of functional activity in the caudate nucleus, a striatal structure that typically shows reduced activity in TTH patients (3). This finding is clinically significant as the caudate nucleus plays a dual role in both nociceptive processing and cognitive-emotional modulation (38). Additionally, acupuncture specifically modulated the parietal operculum, a region crucial for discriminating between self-generated and externally-caused somatosensory information (39). Our study demonstrates that intradermal acupuncture at *Siguan* acupoints can selectively target and regulate precisely those brain regions showing pathological changes in TTH patients which are related to limbic system, including the ACC and caudate nucleus.

### TTH patients and HCs with *Deqi* sensations exhibited inconsistent brain activity changes

In this study, nearly two-thirds of patients with TTH and HCs experienced acupuncture sensations predominantly characterized by acute pain during intradermal acupuncture. These subjects showed functional activity changes in brain regions closely related to pain nociception and processing, such as the precentral/postcentral gyrus, the cuneus/precuneus, and the lingual gyrus. Notably, among those experiencing acute pain sensations, TTH patients exhibited more extensive functional activity changes in brain regions associated with pain processing. However, no specific functional activity changes in brain regions were observed in TTH patients who mainly experienced composite *Deqi* sensations, and HCs experiencing composite *Deqi* sensations only showed functional activity changes in the primary sensory-motor cortex.

In a previous study, it was reported that differences existed in the patterns of activations and deactivations between the grouping of scans associated with *Deqi* sensations versus pain sensations (40). Specifically, compared with the acute pain sensation grouping, the predominately *Deqi* sensation grouping had negative Z-value voxels in the limbic/subcortical structures and the cerebellum regions of interest. Another study investigating the brain functional network of *Deqi* showed that acupuncture modulated the limbic-paralimbic-neocortical network to produce *Deqi* effects (41). However, in this study, we did not observe any specific brain activity changes in patients or healthy individuals with predominant *Deqi* sensation compared to those with predominant pain sensation. That might be caused by the relatively small sample size and the stringent statistic thresholds used in this study.

### Limitations

This study has some limitations. First, we used intradermal needles for the acupuncture intervention. While these needles are convenient for use in an MRI environment and are very commonly used in clinical practice in China, they provide less stimulation intensity compared to traditional acupuncture filiform needles. That might be one important reason why the proportion of patients experiencing *Deqi* sensation was relatively low. Future studies could consider using non-magnetic acupuncture filiform needles for similar research. Second, the subgroup comparisons of *Deqi* sensation were underpowered due to the limited sample size (10 TTH patients versus 11 HCs). While these preliminary findings provide mechanistic insights, future large-scale studies are warranted to confirm the findings. Third, we did not assess long-term therapeutic effects of acupuncture (e.g., duration of headache relief or sustained changes in brain activity), which limits our understanding of the clinical durability of these neuromodulatory effects. Future studies should incorporate longitudinal designs to evaluate both immediate and lasting impacts of acupuncture intervention. Finally, the use of stringent statistical thresholds for fMRI results in this study might have limited the exploration of additional findings.

## Conclusion

The modification of functional activities in the sensorimotor system, DMN and VN is a common brain response characteristic observed in both TTH patients and HCs during acupuncture needling stimulation. In addition, acupuncture at *Siguan* acupoints could extensively regulate the limbic system in TTH patients, and showed targeted modulation in the abnormal brain regions of the ACC and caudate nucleus, which are closely related to the regulation of pain emotions and cognition.

## Data Availability

The original contributions presented in the study are included in the article/supplementary material, further inquiries can be directed to the corresponding authors.

## Footnotes

^1^ http://rfmri.org/dpabi
